# Implementing palliative care in intensive care units: assessing processes using the normalisation process theory NoMAD instrument

**DOI:** 10.1186/s43058-026-00945-8

**Published:** 2026-04-15

**Authors:** Stephanie A. Meddick-Dyson, Tracy Finch, Jason W. Boland, Mark Pearson, Andy Bradshaw, Fliss E. M. Murtagh

**Affiliations:** 1 Wolfson Palliative Care Research Centre, Hull York Medical School, University of Hull, Hull, HU6 7RX UK; 2 Department of Nursing, Midwifery and Health, Northumbria University, Newcastle Upon Tyne, England; 3 Cicely Saunders Institute of Palliative Care, King’s College London, Policy & Rehabilitation, London, UK

**Keywords:** Intensive Care, Palliative Care, Implementation Science, Normalisation Process Theory

## Abstract

**Background:**

The importance of palliative care for Intensive Care Unit (ICU) patients/families is known. Little is known about implementing this care in practice, and how to support healthcare professionals in this implementation. This study uses survey methodology informed by Normalisation Process Theory to assess implementation processes for providing palliative care in the ICU.

**Methods:**

A descriptive cross-sectional survey was conducted with UK healthcare professionals involved in providing or organising palliative care in the ICU. Implementation processes were assessed using the validated 23-item Normalisation MeAsure Development (NoMAD) instrument. Absolute (n) and relative frequencies, median and interquartile ranges were reported. Mann–Whitney U Test assessed differences between specialist palliative care and ICU respondents. One open-ended item captured free-text responses, analysed using NPT-guided framework analysis.

**Results:**

From 153 completed surveys, 69% of respondents were ICU professionals, 31% were specialist palliative care professionals. There was no statistically significant difference between responses from ICU and specialist palliative care professionals. Likert responses showed that respondents felt familiar with palliative care in the ICU and felt it was part of their normal work. Positive tendency was found toward implementation of palliative care in the ICU with coherence (sense-making work), cognitive participation (relational work) and reflexive monitoring (appraisal work). Rating of collective action (operational work) showed a more neutral tendency, highlighting this as a potential target for improvement. Free-text responses were categorised into themes within Normalisation Process Theory constructs: Coherence—recognising and stratifying need, and nuances within palliative care in the ICU; Cognitive participation—interdisciplinary interfaces and building capacity; Collective action—procedures for provision, pressures on provision, and perceived capability; Reflexive monitoring—perceived value.

**Conclusion:**

This novel study uses NPT to assess professional processes relating to implementation of palliative care in the ICU. Findings suggest important perceived implementation gaps may lie within operational work such as tailoring utilisation of existing resources, ensuring leadership support, and building skill sets. Dedicated qualitative research is needed to explain how these issues operate in context and to examine potential patient- and family-related influences.

**Supplementary Information:**

The online version contains supplementary material available at 10.1186/s43058-026-00945-8.

Contributions to the literature

- Informing implementation practice—increasing understanding of how implementation of palliative care in intensive care settings is experienced by providers and organisers and highlighting potential targets for implementation support.
- Methodological demonstration – extending the use of the NoMAD instrument by applying it to palliative care implementation in the ICU, illustrating how Normalisation Process Theory can be operationalised through survey methodology in complex, multidisciplinary clinical settings.
- Identification of knowledge gaps – dedicated qualitative inquiry of palliative care implementation in intensive care settings, inclusive of patient and family perspectives.

## Background

Intensive Care Units (ICUs) deliver specialised care to critically ill patients with life-threatening conditions [1, 2]. Worldwide, the rate of in-hospital mortality for ICU patients is 22% [3] with the majority of these deaths requiring decisions around life-sustaining treatment [4]. Palliative care is an approach that aims to improve quality of life for those facing life-threatening illness by assessing and addressing physical, spiritual, and psychosocial suffering [5]. Physical distress and psychological suffering are commonly reported by ICU patients and their families [6–9]. It is therefore a crucial part of ICU care [10, 11]. Palliative care comprises primary palliative care and specialist palliative care to best make use of resources and respond to needs [12]. Primary palliative care can be delivered by all healthcare professionals and involves provision of aspects of palliative care, such as advance care planning and basic symptom management [12]. Specialist palliative care may involve management of refractory symptoms, existential distress, or complex communication needs [12]. In the UK, specialist palliative care is provided by healthcare professionals who have completed recognised specialist palliative care training and who work within designated specialised palliative care services [13]. This typically includes physicians trained in palliative medicine, specialist palliative care nurses, and allied healthcare professionals with advanced palliative care expertise.

**Fig. 1:**
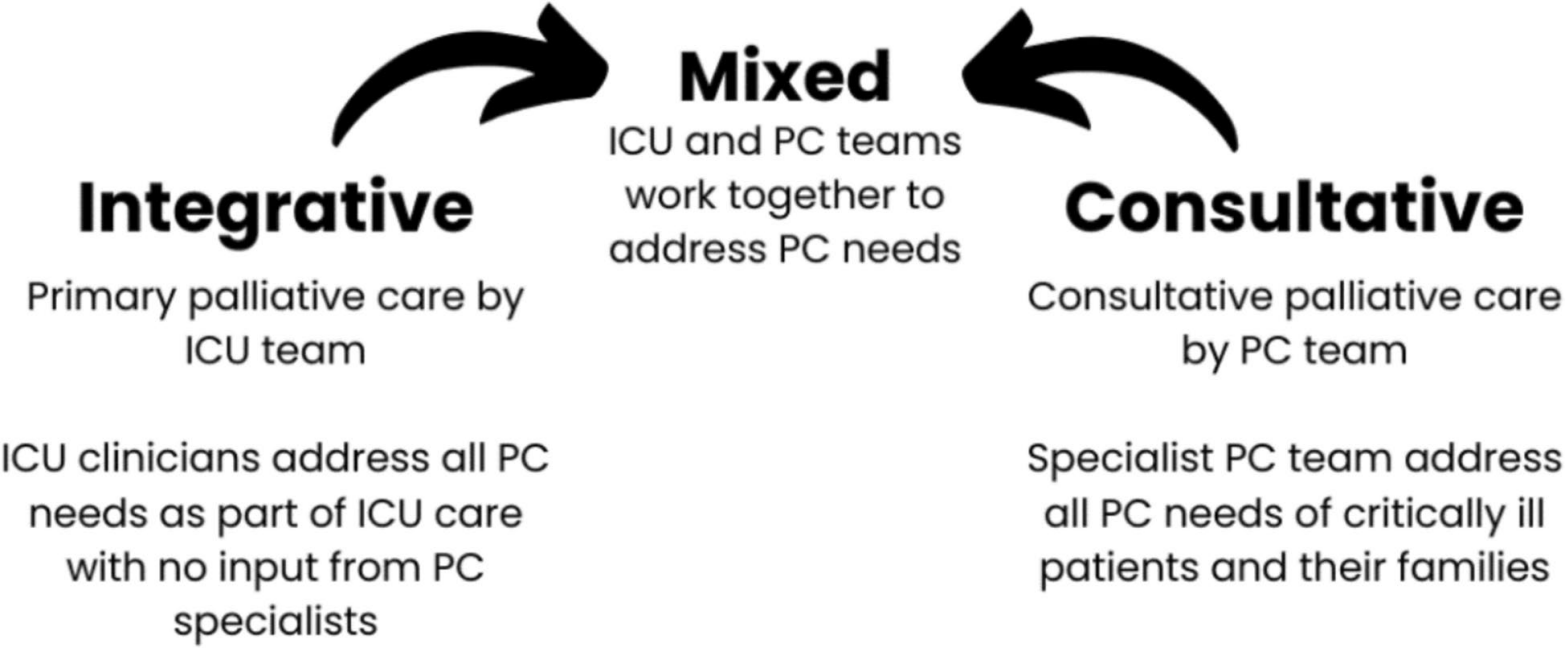
Spectrum of approaches for delivery of palliative care (PC) in the ICU (adapted from Curtis 2022)

Evidence suggests that timely provision of palliative care for ICU patients can reduce unnecessary treatments [14], reduce ICU length of stay without impacting overall mortality [15], and improve perceptions of symptom management [16, 17]. For families, it can reduce stress and increase satisfaction with ICU care and communication [18]. For ICU staff, moral distress can be reduced [19]. There are three main models for provision of palliative care in the ICU: integrative, consultative, or mixed (Fig. 1) [20, 21].

The optimal approach for an individual ICU to provide palliative care will depend on their context, that is, the skill mix and interest of healthcare professionals, patient/family needs, and resource availability [21]. Although intervention studies, for example a complex intervention to improve primary palliative care provided by ICU teams [22] and an intervention to standardise family communication [23], were successful in improving reported quality of death and dying and reducing ICU length of stay and ICU mortality, replication of these interventions in larger, multicentre trials showed no difference in outcomes [24, 25]. This suggests that without consideration of context, interventions may not be transferable. There continue to be proposed opportunities for improvement [21, 26–28] and a focus on principles of *how* palliative care is integrated into ICU care rather than on the form of the care delivered has been recommended [21, 26]. Research calls for a focus on implementation science of palliative care provision in the ICU [29]. Implementation science provides a lens through which we can use validated tools and frameworks to enact these recommendations. Implementation strategies refer to “methods or techniques used to enhance the adoption, implementation, and sustainability” of an evidence-based intervention [30, 31]. Determinants are the modifiable factors that the implementation strategy aims to change to influence implementation of evidence-based interventions [31, 32], in other words, factors that facilitate, or provide barriers to, implementation. Mechanisms are the events or processes through which implementation strategies work [33].

A systematic review of studies examining healthcare professional perceptions of factors influencing a palliative care approach in the ICU setting suggested that both organisational factors (e.g. resource and time constraints), and individual factors (healthcare professional, patient, and family attitudes, communication, skillset and knowledge) can hinder the integration of a palliative care approach in the ICU context [34]. Two more recent scoping reviews reported barriers to (lack of skillset, family boundaries, cultural differences, and practical issues) and facilitators for (collaboration and adequate communication between teams and with family members, experience in providing palliative care, and adequate training and education) providing palliative care in the ICU [35, 36]. Prior to this survey, authors conducted a systematic review using a modified implementation research logic model (IRLM) [37] to identify, synthesise, and map evidence on implementation of (primary and/or specialist) palliative care in the ICU [38]. Rather than healthcare professional perceptions, this review synthesised implementation factors (strategies, determinants and mechanisms) reported within trials of palliative care interventions in the ICU, and process evaluations. Whilst there were again common barriers (lack of resources, negative perceptions of palliative care, and high ICU acuity) and facilitators (adequate resources and collaboration between palliative care and ICU teams) when implementing palliative care in the ICU, mechanisms were rarely reported. As evidenced by these studies, although necessary to provide a common and transferable taxonomy, implementation frameworks are by nature descriptive [39]. Given the need for context-specific, tailored, strategies for individual ICUs [21, 38], it is vital that we seek to understand the process behind *how* these determinants and strategies work. For palliative care in the ICU, these implementation processes are those through which palliative care is embedded into everyday work and sustained (integrated) in practice. Given the importance of collaboration, assessment of how experiences of implementation may compare between healthcare professionals who provide primary and specialist palliative care is a crucial part of this understanding. Implementation theory, specifically Normalisation Process Theory (NPT), provides the explanatory power for this goal with its conceptual framework of core types of work that make up these processes: coherence, cognitive participation, collective action, and reflexive monitoring (Table 1) [40]. NPT has been used effectively to guide and understand intervention development and implementation in other palliative care contexts [41, 42]. It facilitated identification of mechanisms and relationships underpinning successful implementation of person-centred outcome measures in palliative care into routine practice [41], and the individual, organisational, and technical factors influencing the implementation of the Electronic Palliative Care Coordination Systems [42].

**Table 1:** NPT constructs described and applied to palliative care in the ICU. Derived from May et al. (2009) [45]

Construct	Generalised Definition	Within the study context
Coherence	Sense-making work that individuals or organisations do when they are tasked with implementing a complex intervention or set of practices	Making sense of what palliative care approaches/interventions are within the ICU, and how/when to provide them
Cognitive participation	Relational work that people do to engage with a complex intervention or set of practices, to build and sustain a community of practice around it	What is done to engage with palliative care approaches/interventions within the ICU, to build and sustain a community around provision
Collective action	Operational work that people do to enact a complex intervention or set of practices	The ways in which individuals or organisations work independently or collectively to provide palliative care approaches/interventions within the ICU
Reflexive monitoring	Appraisal work that people do to assess and understand how a complex intervention or set of practices affect(s) them and others around them	How people appraise and assess the value and use of palliative care approaches/interventions within the ICU

### Aim

The aim of this study is to assess implementation processes for providing palliative care in the ICU using survey methodology informed by NPT. This adds to the current literature by providing insight into perceived strengths and gaps of implementation work and potential key mechanisms that underpin implementation of palliative care in the ICU. This new insight can help identify targets for implementation support for interventions and approaches that aim to facilitate and improve palliative care provision within ICU settings.

## Methods

### Design

Descriptive, cross-sectional (administered to each participant once at one time point), national online survey with an optional open-ended item hosted on the Qualtrics platform via a University of York secure account.

### Guiding theoretical framework

Normalisation Process Theory (NPT) is an implementation theory that supports the development, evaluation, and implementation of complex interventions [40]. May et al. systematically reviewed the theory’s use in feasibility studies and process evaluations of complex healthcare interventions. They found the theory was effectively used across a wide range of interventions to aid development and implementation planning, as well as gaining an understanding of the implementation processes [43]. NPT provides a set of tools based in sociological science to understand and explain the processes surrounding implementation of practices within a given context [44, 45]. For these reasons, it was identified as a fitting theory to inform this work and provide the theory to build on the descriptive outputs from the previous systematic review.

Consisting of four constructs, it offers a way to describe the ‘work’ required for organisations and individuals to ‘normalise’ palliative care (i.e. implement) into everyday routine. Table 1 explains these constructs and how they translate to this study.

The Normalisation MeAsure Development (NoMAD) instrument, used in this study, is a validated survey providing a quantitative measure of the NPT constructs [46, 47]. It was developed to assess implementation processes and understand implementation participants’ experiences [47]. The survey items have been refined using psychometric analysis and the instrument validated with a sample of 831 respondents over six different implementation projects [48].

### Participants and setting

A convenience sample of healthcare professionals was recruited through targeted social media advertisements and membership groups (Intensive Care Society, Association for Palliative Medicine, End-of-life and Palliative Care in the ICU Research Network). ICU and specialist palliative care (SPC) professionals who self-reported as having experience of providing or organising palliative care provision within an ICU in the United Kingdom (UK), were 18 years or older, and who were able to consent to and complete the survey, were eligible to take part. Participants were asked to self-identify whether being part of the ICU team or part of the palliative care team (SPC) best described their role. If respondents had experience as part of the ICU team and the palliative care team, they were asked to select one role and answer in relation to that experience.

### Data collection

The survey ran between February and April 2023. Pre-set questions from the NoMAD instrument were used, tailored to ask about the processes by which healthcare professionals work to provide palliative care in their ICU (Supplementary Material). The NoMAD instrument is a 23-item survey that directly measures the implementation process of a complex intervention, or category of interventions, from the perspective of those directly involved in the work of implementation [49]. The instrument is designed such that the intervention under exploration can be inserted into the questions. For this study, “providing palliative care in the ICU” was used. Tailoring the instrument to explore implementation in this way is the intended use by its creators [49]. Initial screening questions checked eligibility as an ICU or palliative care professional with experience delivering or organising palliative care within the ICU in the UK before completing the survey. The first three items explore the respondents’ experience and expectations of the implementation process. These are rated using a 0–10 scale [49]. Each of the four NPT constructs then have statements presented as Likert items where respondents are asked to indicate their level of agreement or select “not relevant” [49].

The final survey was a multiple-choice survey with a single open-ended item, “Would you like to tell us anything else about how you work to provide palliative care in your intensive care unit?” to offer supplementary comments regarding implementation of palliative care in the ICU (supplementary material). This open-ended item collected short-form qualitative data to complement quantitative findings [50]. It was piloted with healthcare professionals from SPC and ICU backgrounds.

Qualtrics allows one entry per participant. Ethical approval of the protocol and data management plan was given by Hull York Medical School Ethics Committee (22–23 21).

### Analysis

Only completed surveys were included in analysis. Demographic information is presented in summary. For analysis and interpretation, Likert responses were coded to 1 = strongly disagree to 5 = strongly agree. Higher scores therefore represent higher agreement. Although the initial general questions use a 1–10 scale, only whole numbers can be selected, therefore this data is treated as ordinal data, as with the individual Likert items. Absolute (n) and relative frequencies for each score are reported, and the median and interquartile range for each question. Divergent bar charts allow visualisation of the data spread. For each statement, Mann–Whitney U Test was used to report whether there was a statistical difference between responses from SPC professionals and ICU professionals. *U *(*N*_ICU_ = 105, *N*_SPC_ = 48), *z *score, and *p *value are presented for each statement. “Not relevant” responses are not shown in this divergent bar chart as this is demonstrating directional spread of responses.

Free-text responses to the single open-ended survey item were analysed thematically, guided by the four NPT constructs as a framework; coherence, cognitive participation, collective action, and reflexive monitoring (as described in Table 1). Steps of framework analysis outlined by Ritchie and Spencer were followed; familiarisation, identifying a framework, indexing, charting, and mapping and interpretation [51]. Responses were read multiple times (familiarisation), NPT was deemed an appropriate framework (identifying a framework), responses were coded deductively according to NPT constructs through formulation of a structured categorisation matrix (indexing, charting, and mapping) and summarised to illustrate key patterns (interpretation). Analysis was led by SMD and reviewed by co-authors.

Integration of quantitative and qualitative data occurred at the interpretation stage. Free-text responses were used to complement and contextualise quantitative findings, offering narrative context to support interpretation of quantitative trends.

## Results

### Respondents

There were 153 completed surveys. 69% (N = 105) of respondents were ICU professionals and 31% (N = 48) were SPC professionals. Table 2 shows the demographic spread.

**Table 2:** Respondent demographics

	**Number(%)** **N = 153**		**Number(%)** **N = 153**
**Age**		**Location within the UK**	
18–29 years	12(8)	East Midlands	7(5)
30–39 years	44(29)	East of England	4(3)
40–49 years	57(37)	Kent, Surrey and Sussex	4(3)
50–59 years	31(20)	London	17(11)
60–69 years	6(4)	North East, including north Cumbria	14(9)
Prefer not to say	3(2)	North West	6(4)
**Gender**		Northern Ireland	13(8)
Male	49(32)	Scotland	27(18)
Female	98(64)	South West	23(15)
Non-binary/third gender	1(1)	Thames Valley	3(2)
Prefer not to say	5(3)	Wessex	5(3)
**Ethnicity**		West Midlands	8(5)
Asian or Asian British	12(8)	Yorkshire and the Humber	18(12)
Black, Black British, Caribbean or African	5(3)	Prefer not to say	4(3)
		**Professional group**	
Mixed or multiple ethnic groups	11(7)	Specialist palliative care	48(31)
Other ethnic group	4(3)	Intensive care	105(69)
White	114(75)		
Prefer not to say	7(5)		

### Quantitative findings

**Fig. 2:**
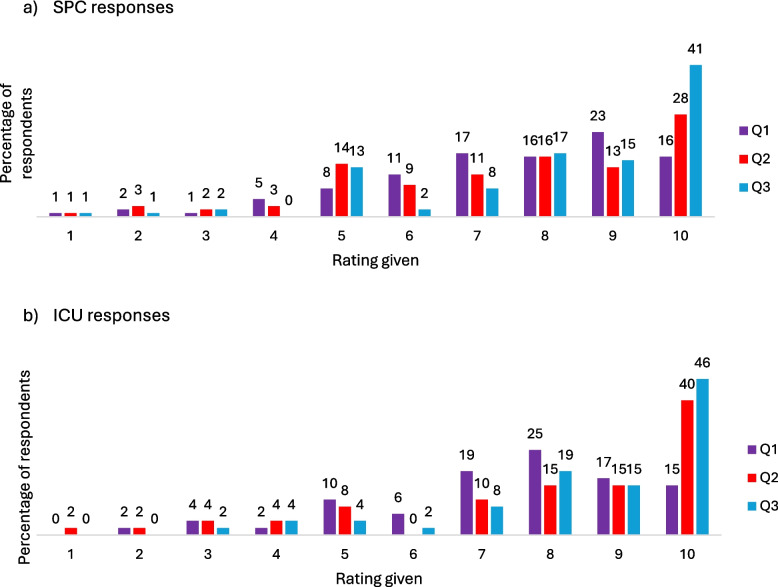
Percentage distribution of ratings for initial questions by **a** SPC respondents and **b** ICU respondents

The first three items of the NoMAD instrument explore the respondents’ experience and expectations of the implementation process (Table 3 and Fig. 2). Responses were similar between groups and overall responses suggest a high level of familiarity and that providing palliative care in the ICU is both part of their normal work, and even more so will become a normal part of their work.

**Table 3:** Median and interquartile ranges for items exploring respondents’ experience and expectations of the implementation process

Question	Median (Interquartile Range)		
	Combined (N = 153)	SPC professionals (N = 48)	ICU professionals (N = 105)
1. When you are involved in providing palliative care in the ICU, how familiar does it feel? *0 (still feels very new) to 10 (feels completely familiar)*	8(3)	8(2)	8(3)
2. Do you feel providing palliative care in the ICU is currently a normal part of your work? *0 (not at all) to 10 (completely)*	8(4)	9(3)	8(4)
3. Do you feel providing palliative care in the ICU will become a normal part of your work in the future? *0 (not at all) to 10 (completely)*	9(2)	9(2)	9(3)

Table 4 shows the absolute frequencies, percentages, medians, interquartile ranges including “Not relevant” responses, and Mann–Whitney U Test statistics of the responses. For analysis and interpretation, Likert responses were coded to 1 = strongly disagree to 5 = strongly agree and treated as ordinal data. Of note, there is a question within collective action “providing palliative care in the ICU disrupts working relationships” where negative rating represents a positive response. Median scores for NoMAD items within coherence and cognitive participation were either 4 or 5 for combined responses (N = 153). Median scores for reflexive monitoring were 4 or 5 except for the statement “I am aware of reports about the effects of providing palliative care in the ICU” where the median score was 3. Median scores for collective action were more commonly 2 or 3. Mann–Whitney U Testing found no significant difference (p > 0.05) between responses from each team for any of the NoMAD statements.

**Table 4:** Absolute frequencies, percentages medians, interquartile ranges, and Mann–Whitney U test statistics of the responses

	Strongly agree n(%)		Agree n(%)		Neither agree nor disagree n(%)		Disagree n(%)		Strongly disagree n(%)		Not relevant n(%)		Median (Interquartile Range)		Mann–Whitney U Test		
	ICU N = 105	SPC N = 48	ICU N = 105	SPC N = 48	ICU N = 105	SPC N = 48	ICU N = 105	SPC N = 48	ICU N = 105	SPC N = 48	ICU N = 105	SPC N = 48	ICU N = 105	SPC N = 48	*U* *N*_*ICU*_ = 105 *N*_*SPC*_ = 48	z-score	p-value (0.05)
**Coherence**																	
I can see how providing palliative care in the ICU differs from usual ways of working	28(27)	12(25)	52(50)	22(46)	9(9)	2(4)	10(10)	9(19)	6(6)	3(6)	0(0)	0(0)	4(1)	4(2)	2363	0.615	0.535
Staff in this organisation have a shared understanding of the purpose of providing palliative care in the ICU	18(17)	4(8)	48(46)	22(46)	20(19)	7(15)	17(16)	12(25)	2(2)	2(4)	0(0)	1(2)	4(1)	4(2)	2101	1.646	0.099
I understand how providing palliative care in the ICU affects the nature of my own work	27(26)	7(15)	59(56)	31(65)	14(13)	5(10)	4(4)	2(4)	0(0)	1(2)	1(1)	2(4)	4(1)	4(0)	2212	1.209	0.226
I can see the potential value of providing palliative care in the ICU for my work	66(63)	33(69)	34(32)	13(27)	3(3)	1(2)	1(1)	0(0)	0(0)	1(2)	1(1)	0(0)	5(1)	5(1)	2373.5	−0.574	0.567
**Cognitive participation**																	
There are key people who drive providing palliative care in the ICU forward and get others involved	26(25)	7(15)	49(47)	23(48)	20(19)	14(29)	10(10)	3(6)	0(0)	1(2)	0(0)	0(0)	4(1)	4(1)	2192.5	1.286	0.197
I believe that participating in providing palliative care in the ICU is a legitimate part of my role	77(73)	32(67)	27(26)	15(31)	1(1)	0(0)	0(0)	0(0)	0(0)	1(2)	0(0)	0(0)	5(1)	5(1)	2345.5	0.684	0.497
I’m open to working with colleagues in new ways to provide palliative care in the ICU	78(74)	32(67)	25(24)	14(29)	1(1)	0(0)	0(0)	0(0)	0(0)	0(0)	1(1)	2(4)	5(1)	5(1)	2316	0.800	0.424
I will continue to support providing palliative care in the ICU	81(77)	34(71)	21(20)	12(25)	0(0)	0(0)	0(0)	0(0)	0(0)	0(0)	3(3)	2(4)	5(0)	5(1)	2358	0.635	0.522
**Collective action**																	
I can easily integrate providing palliative care in the ICU into my existing work	40(38)	18(38)	51(49)	22(46)	8(8)	1(2)	4(4)	6(13)	1(1)	1(2)	1(1)	0(0)	4(1)	4(1)	2434.5	0.334	0.741
Providing palliative care in the ICU disrupts working relationships	0(0)	0(0)	2(2)	2(4)	14(13)	3(6)	34(32)	19(40)	55(52)	24(50)	0(0)	0(0)	1(1)	2(1)	2516	−0.014	0.992
I have confidence in other people’s ability to provide palliative care in the ICU	14(13)	5(10)	52(50)	26(54)	21(20)	9(19)	17(16)	4(8)	1(1)	3(6)	0(0)	1(2)	4(1)	4(1)	2476	0.171	0.865
Work is assigned to those with skills appropriate to provide palliative care in the ICU	9(9)	3(6)	40(38)	22(46)	38(36)	14(29)	14(13)	8(17)	3(3)	1(2)	1(1)	0(0)	3(1)	4(1)	2458	−0.242	0.810
Sufficient training is provided to enable staff to implement providing palliative care in the ICU	3(3)	1(2)	23(22)	16(33)	22(21)	9(19)	48(46)	14(29)	7(7)	7(15)	2(2)	1(2)	2(1)	3(2)	2375	−0.568	0.569
Sufficient resources are available to support providing palliative care in the ICU	12(11)	2(4)	31(30)	16(33)	19(18)	13(27)	34(32)	14(29)	6(6)	2(4)	3(3)	1(2)	3(2)	3(2)	2498	0.085	0.936
Management adequately supports providing palliative care in the ICU	8(8)	4(8)	41(39)	17(35)	34(32)	22(46)	14(13)	1(2)	3(3)	3(6)	5(5)	1(2)	3(1)	3(1)	2440.5	−0.311	0.757
**Reflexive monitoring**																	
I am aware of reports about the effects of providing palliative care in the ICU	13(12)	2(4)	35(33)	17(35)	19(18)	5(10)	31(30)	18(38)	6(6)	5(10)	1(1)	1(2)	3(2)	3(2)	2126.5	1.545	0.121
The staff agree that providing palliative care in the ICU is worthwhile	39(37)	11(23)	56(53)	30(63)	9(9)	5(10)	1(1)	0(0)	0(0)	1(2)	0(0)	1(2)	4(1)	4(0)	2108	1.618	0.105
I value the effects that providing palliative care in the ICU has had on my work	49(47)	17(35)	51(49)	28(58)	4(4)	1(2)	1(1)	1(2)	0(0)	0(0)	0(0)	1(2)	4(1)	4(1)	2226	1.154	0.250
Feedback about providing palliative care in the ICU can be used to improve it in the future	61(58)	26(54)	42(40)	20(42)	1(1)	1(2)	1(1)	0(0)	0(0)	1(2)	0(0)	0(0)	5(1)	5(1)	2398.5	0.476	0.631
I can modify how I work to provide palliative care in the ICU	30(29)	14(29)	65(62)	26(54)	6(6)	6(13)	0(0)	0(0)	0(0)	2(4)	4(4)	0(0)	4(1)	4(1)	2415	0.411	0.682

**Fig. 3:**
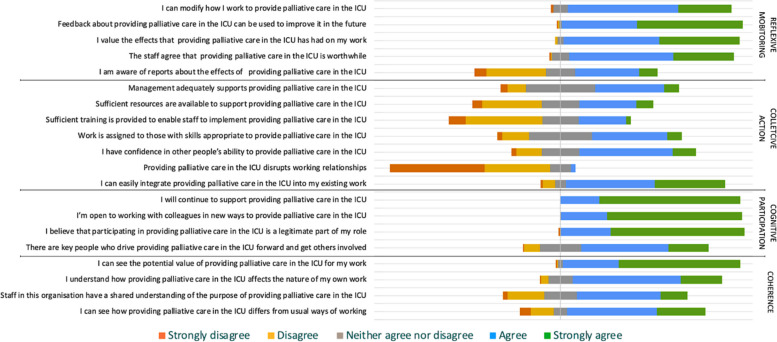
Divergent bar chart illustrating the spread of answers for each NoMAD statement on providing palliative care in the ICU. The grey bars spanning the midline represent “neither agree nor disagree”. Answers to the left of the midline indicate negative reporting (“disagree/strongly disagree”) and answers to the right, positive reporting (“agree/strongly agree”)

Figure 3 is a divergent bar chart illustrating the spread of answers for each NoMAD statement on providing palliative care in the ICU from all completed responses (N = 153). This demonstrates a visual representation of the positive or negative reporting for each statement, enabling identification of areas for improvement. Responses related to collective action (operational work) are notably more negative.

### Free-text responses

41 respondents gave an optional free-text answer to the question “would you like to tell us anything else about how you work to provide palliative care in your intensive care unit?”. 12 of these were SPC professionals, 29 ICU professionals. Thematic framework analysis corresponding to the four NPT constructs (as described in Table 1) is presented below.

#### Coherence: making sense of what palliative care approaches/interventions are within the ICU, and how/when to provide them

##### Theme 1 – Recognising and stratifying need

Subtheme – Complexity

Palliative care was discussed as a routine part of ICU care; however, cases were noted where SPC input was needed.

ICU professionals described palliative care and recognition of palliative care need as a “common and routine” (respondent 19, ICU), “integral and crucial” (respondent 35, ICU), “very important” (respondent 85, ICU), and “inevitable” (respondent 43, ICU) part of ICU care, and “as much part of ICU as inotropes or ventilation” (respondent 71, ICU).

Both SPC and ICU professionals reported that SPC input was sought for complex patients. This included those who were awake rather than unconscious, required complex decision making, and this complexity was contrasted to withdrawal of life sustaining treatment in “straight forward patients” (respondent 98, ICU). It was discussed how death for these patients on full support usually follows quickly and that “patient on minimal support who are more ward based may get a poorer deal” (respondent 28, ICU)”. Other reported needs were “symptom control”, and “patient and carer support and during the transition to a ward where appropriate” (respondent 27, SPC). The recognition of need in patients who are “usually compromised by the nature of admitting patients requiring organ support” (respondent 104, ICU) was said to be challenging.

Subtheme – The dichotomy between ICU and palliative care

The conflation of palliative care with end-of-life care was noted, and a focus of ICU palliative care on patients for whom life sustaining treatment is withdrawn. There was a described dichotomy between ICU and palliative care with the terms “moving to palliative” (respondent 85, ICU) and “switching to palliative care” (respondent 87, ICU) used, and an “Inherent tension between aggressive intensive care and palliative care” (respondent 117, ICU). Respondents reported a lack of a “dual approach” (respondent 52, ICU), or “parallel planning” (respondent 68, ICU) with nurses “more open to palliative care and earlier in the dying trajectory than consultants” (respondent 2, ICU).

##### Theme 2 – Nuances within palliative care in the ICU

The “*nuances of palliative care for ICU patients”* were apparent with ICU professionals acknowledging their ICU specific expertise were important. Small group training interventions were felt to be “not suitable to the ICU environment as inevitably it will be a member of staff caring for the patient that has not had that training” (respondent 132, ICU). Lack of understanding by the SPC team of these nuances was felt to be an issue by one ICU professional. Contradictory to this, one SPC professional described the need to tailor the use of medications to intravenous routes due to ICU team familiarity with this route.

#### Cognitive participation: what is done to engage with palliative care approaches/interventions within the ICU, to build and sustain a community around provision

##### Theme 3 – interdisciplinary interfaces

Subtheme – Collaborations

The close working interface and “very good collaborative working” (respondent 32, ICU) between the SPC team and the ICU team was communicated, including their support with symptom support, patient and carer support, and complex patients as outlined above. A “network of champions” (respondent 109, SPC) was described working within ICU who link to the SPC team, as well as “End-of-life care leads” (respondent 17, ICU) and a “care at end of life team” (respondent 141, ICU). The procedural practicality of the SPC involvement is discussed within *Collective Action*. It is pertinent to note that one SPC professional felt that they “do not have the capacity to be pro-active, it is the ITU consultant who decides when to refer to palliative care” (respondent 1, SPC).

Collaboration was also apparent amongst the ICU team, through a “multi-disciplinary approach” (respondent 17, ICU), and an “ethos to pull together to ensure the best care” (respondent 80, ICU now SPC). Here, the leading role of the ICU consultant around palliative care was evident.

Subtheme – Conflict

The tension between intensive and palliative care was noted in *Coherence.* There was also mention of “pushback from the parent team (e.g. surgery, medical, haematology) about even involving SPC teams” (respondent 68, ICU). Within ICU, respondents communicated a “disparity between nurses and medics” (respondent 1, ICU) with regards to timing of introducing palliative care, and the provision of the care itself.

Subtheme—Team dynamic

Both respondents from the ICU and SPC team perceived that palliative care provision can be impacted by “team dynamic on the day” (respondent 132, ICU), and “the culture and skill level of the ICU team at the time” (respondent 112, SPC).

##### Theme 4 – building capacity

One respondent described a “huge shift in the nursing skill mix” and that the “general pool of nurse working in ICU are junior nurses” (respondent 80, ICU to SPC). The SPC described involvement in “teaching to nursing staff in ICU on palliative and end of life” (respondent 27, SPC). However, ICU respondents felt training “is often less prioritised than other areas” (respondent 39, ICU) and its limitation to small numbers of staff is insufficient. Having guidance in place was felt to be “greatly beneficial to those less comfortable with end-of-life management” (respondent 44, ICU). Although it was also stated that despite familiarity through experience, ICU staff “probably don't have a formalised or structured framework on how they would approach this” (respondent 86, ICU).

#### Collective action: the ways in which individuals or organisations work independently or collectively to provide palliative care approaches/interventions within the ICU

##### Theme 5—procedures for provision

The sense that palliative care is an integral role within the ICU, the importance of collaboration, and the challenges of conflict and dichotomous thinking are presented earlier. Comments also alluded to the procedural aspects of palliative care provision.

Communication with the SPC was through referral systems, champions within the ICU, or regular discussion through meetings or ward rounds and collaboration was described as “on a case-by-case basis as to what is required and why” (respondent 140, SPC). To add to this, “lots of consultant-to-consultant discussions and joint family meetings” (respondent 25, SPC) between ICU and SPC were noted. Some respondents reported that it is ICU consultants who decide who to refer to the SPC as well as provide the care, and they are “very capable of delivering this (at) a decent level” (respondent 16, ICU). However, others felt delivery of palliative care was part of the nursing role, offering “hope, comfort and pragmatism” (respondent 114 ICU), and that “more than probably should be in terms of communication and management of palliative care is left to the bedside nurse” (respondent 35. ICU). There were also comments suggesting lack of confidence in the medical teams’ approach to palliative care in terms of delayed recognition of need, the lack of parallel planning, and overmedicalisation.

##### Theme 6 – pressures on provision

The acuity of the ICU provided time pressures as well as difficulties with recognising palliative care need. “Organisational pressure” (respondent 85, ICU) was recognised with regards to ICU beds as valuable resources, and the pressure of time was referenced. One respondent specifically mentioned the organisational challenges involved in enabling patients who wish to die at home to do so.

##### Theme 7—perceived capability

Respondents reflected on their capability regarding palliative care. There were mixed views as to whether recognition and management of need was adequate. Some respondents praised these aspects, but others highlighted areas for improvement in terms of parallel planning, timely recognition, symptom management for longer-term patients, and end-of-life practices. For example, one respondent explained how the “Medical team regularly want to keep monitoring on patient which is often distressing for family to see and not comfortable for patient” (respondent 52, ICU). The pressures discussed above were acknowledged to impact these capabilities.

#### Reflexive monitoring: How people appraise and assess the value and use of palliative care approaches/interventions within the ICU

##### Theme 8—perceived value

Respondents suggested that a good ICU can provide palliative care for its patients and that “death is not a failure” (respondent 64, ICU). They recognised some of the benefits to include “a dignified, non-medicalised death” (respondent 43, ICU), and “hope, comfort and also pragmatism” (respondent 114, ICU). Comments highlighted the SPC adding value and reflected on collaborative work to be “amazing” (respondent 45, SPC). For staff, as previously mentioned, guidance was felt to be beneficial. Despite palliative care proving challenging at times, it was said to be “very rewarding for all involved” (respondent 107, ICU). Measurement of impact was only mentioned in one comment referring to a Quality Improvement project that had “highlighted the importance of integrating pall(iative) care and ITU” (respondent 8, SPC).

## Discussion

### What this study adds

Tailoring the validated NoMAD instrument, as suggested by the creators [52], to providing palliative care in the ICU allowed assessment of implementation processes using NPT. By consulting SPC and ICU professionals, it allowed exploration of potential differences between the two groups.

Answers to the initial three questions, exploring experience and expectations of the implementation process, were similar between ICU and SPC professionals. SPC professionals gave a slightly lower rating for question 1 referring to familiarity. However, they gave higher ratings regarding palliative care in the ICU being a normal part of their work. Interestingly, the free-text comments indicated that ICU professionals do feel palliative care is a crucial part of their role whereas there were no comments around this from SPC professionals. Both teams could describe the roles of the SPC team and when they were needed within their free-text comments. They suggested that within the ICU, consultants had a leading role, with consultants and nursing staff delivering palliative care. Involving ICU nurses in the provision and leadership of palliative care interventions did help with compatibility in the ICU setting [38].

The patterns seen within statement ranking were consistent between ICU and SPC professionals. Visually, Figs. 2a and 2b are almost identical and Mann–Whitney U Testing found no significant difference (p > 0.05) between responses from each team for any of the NoMAD statements (Table 4). This finding of a shared understanding is promising for the future of palliative care in the ICU, with evidence suggesting that a symbiotic relationship between ICU and SPC teams is a common facilitator [35, 38].

Positive tendency for responses related to coherence suggests that ICU and SPC professionals feel they can make sense of what palliative care within the ICU is, and how and when to provide it. Respondent comments spoke to the important nuances of palliative care in the ICU and highlighted a perception of a dichotomy between ICU and palliative care both as an area for improvement and a source of potential conflict. Tension for change and perceived benefit/relative advantage have been found to act as facilitators when present, and barriers when absent [38]. Strategies such as conducting a local needs assessment, having SPC professionals on the ICU ward round, and providing the evidence base for an intervention, work to utilise this by demonstrating a need for palliative care in the ICU [38]. Both teams were able to describe the situations that benefit from SPC team involvement. However, comments echoed the literature finding that decision making and prognostication can present a barrier to integrating palliative care in ICUs [34]. One strategy to counteract this is for an intervention to be applicable for all ICU patients from the start of the admission [38]. Comments emphasising palliative care as an integral part of ICU care concur with this.

Positive tendency in responses for cognitive participation suggests work is being done to engage with providing palliative care within the ICU, to build and sustain a community around provision. This relates to both teams feeling that providing palliative care in the ICU is part of their role. It is reflected in the comments around the importance of collaboration between ICU and SPC teams. Effective multidisciplinary team working and collaboration is a well-documented facilitator for palliative care in the ICU [34, 35, 38]. Strategies to maximise this include clearly defining roles, interprofessional learning at morbidity and mortality reviews, and creation of a palliative care in the ICU workgroup [38]. Collaboration with family members is discussed within the literature as facilitator [34, 36, 38], and lack thereof a barrier [35, 38]. Family meetings were mentioned in one free-text comment but in relation to professional roles. Otherwise, patient and family factors were not discussed.

Responses to statements related to reflexive monitoring suggest that ICU and SPC professionals positively appraise and assess the value and use of palliative care provision within the ICU. Comments echoed this finding but only once was a formal assessment of value (through Quality Improvement) mentioned. In keeping with this, although participants tended to agree that feedback can be used to improve palliative care in the ICU, responses to the statement “I am aware of reports about the effects of providing palliative care in the ICU” trended more negatively than the other statements in this construct. In the literature, providing staff with formal feedback facilitated physician buy-in and changes for improvement [38]. This suggests a call for use of audit and feedback style approaches as a potential implementation strategy, although it is reassuring that free-text comments suggest a perceived benefit without awareness of evidence. Inclusion of a question relating to participants’ awareness of informal feedback about the effects of providing palliative care in the ICU, in addition to the more formally termed ‘reports’, may allow the NoMAD instrument to explore this discrepancy.

Statements relating to collective action, the ways in which individuals or organisations work independently or collectively to provide palliative care within the ICU, showed a more neutral tendency. Most notably, the statements asking about resources, training and managerial support were more negatively rated than other statements. This concurs with free-text comments made around the pressures of ICU and lack of training. In line with this, inadequate resources and training are other well documented barriers to palliative care in the ICU [34–36, 38]. Healthcare professional experience has been reported as a facilitator of provision [35]. Strategies to optimise resources include involving ICU leadership and supporting ICU teams to provide primary palliative care themselves [38]. Short and frequent training sessions, use of spontaneous learning opportunities, and expert ward rounds have been used as educational strategies [38]. One comment in this study suggested that it is important for training to reach all staff. Formulation of policies and guidelines, as suggested in the free-text comments, is also recommended in the literature [34, 36, 38]. Of note, there is a question within collective action “providing palliative care in the ICU disrupts working relationships” where negative rating represents a positive response (i.e. it does not disrupt). The positive response found in the study is in keeping with reported perceptions in free-text comments of palliative care being integral to ICU care and important collaborations within and between teams. Cultural differences can hinder palliative care provision in the ICU [35, 38]. The NoMAD instrument does not directly ask about culture, but one may expect a more negative response for this statement if that were felt to be the case here.

### Strengths and limitations

These findings imply that ICU and SPC professionals make sense of palliative care in the ICU, it fits within their (sense of their own) professional roles, they feel able to participate, and perceive benefit. Similarities in their responses is a positive signal for a shared understanding and drive for improvement. However, processes and logistical difficulties may be inhibiting optimal provision. This is a common finding in NPT research [53, 54]. Both aspects of this study suggest that important implementation gaps may lie within operational work. In this context, this is *the ways in which individuals or organisations work independently or collectively to provide palliative care approaches/interventions within the ICU*. Investing in addressing the operational issues to better support collaborative working in the ICU may represent a promising target for implementation support.

Strengths of this work include the use of a validated instrument. Respondents to this survey span the UK and give representation from the ICU and SPC communities although authors recognise the overrepresentation of female respondents. This may allow for naturalistic generalisation to other healthcare professionals working to provide palliative care in the ICU in that findings resonate with their lived experiences. However, free-text comments cannot be said to be representative of the sample and should not be generalised.

Whilst use of the NoMAD instrument provides theory-driven insights into implementation processes, its exploratory and explanatory power is limited when compared to that dedicated qualitative approaches. While free-text responses in surveys lack the depth and dialogical richness of data generated through methods such as semi-structured interviews, they nonetheless represent a recognised and valid source of qualitative insight suitable for thematic, content, or narrative analysis [55]. The use of free-text responses within the NoMAD instrument in this way has been previously demonstrated [56]. In this study, despite the limited volume and depth of responses, the qualitative data provided illustrative insights into how healthcare professionals enact and perceive the processes measured by the survey. This reflects the principle of complementarity; whereby qualitative data enrich quantitative findings by revealing practical nuances not captured by closed-ended items [50]. For example, the Family Evaluation of Hospice Care (FEHC) survey similarly incorporates free-text responses to acknowledge the complexity of palliative care and the value of narrative in deepening understanding of structured data, as well as the benefit of the storytelling opportunity for respondents [57]. Finally, it is important to consider patient- and family-related factors that may influence provision of palliative care in the ICU.

### Future directions

Future work considering palliative care in the ICU should therefore focus on developing resources and training to support palliative care provision and navigating the complex, but vital, interplay between multidisciplinary teams. Exploration and explanation of patient- and family-related influences on palliative care provision in the ICU is needed to ensure these are considered within future implementation strategies. Robust qualitative investigation using the NPT constructs with SPC and ICU health professionals would enable collection of more explanatory data to guide these resources. For the wider field of palliative care research, NPT provides a structure for exploring implementation processes behind palliative care provision in other complex healthcare contexts, or those behind the use of specific palliative care interventions. Using this structure provides actionable outputs that can guide future implementation efforts. The NoMAD instrument offers a practical way to gather these insights. This study saw the benefits of providing space for free-text responses to supplement quantitative findings, but using NPT to guide qualitative inquiry through interviews or focus groups would allow richer data collection.

## Conclusion

This novel study uses NPT to assess professional processes relating to implementation of palliative care in the ICU. Findings suggest important perceived implementation gaps may lie within operational work such as tailoring utilisation of existing resources, ensuring leadership support, and building skill sets. Dedicated qualitative research is needed to explain how these issues operate in context and to examine potential patient- and family-related influences.

## Supplementary information

Supplementary Material 1.

## Data Availability

Tailored NoMAD instrument available in supplementary material.

## Abbreviations

ICU: Intensive Care Unit
NoMAD: Normalisation MeAsure Development
NPT: Normalisation Process Theory
PC: Palliative Care
SPC: Specialist Palliative Care
UK: United Kingdom
