# Supplementary material for: Implementing palliative care in intensive care units: assessing processes using the normalisation process theory NoMAD instrument
Source: Implement Sci Commun. 2026 Apr 15;7:103. doi: 10.1186/s43058-026-00945-8 (PMC13195999; doi:10.1186/s43058-026-00945-8)
Supplement: Supplementary file 1 — Supplementary Material 1. [file 43058_2026_945_MOESM1_ESM.docx]

**Supplementary Material: Adapted Normalisation MeAsure Development (NoMAD) survey**

**Pre-screening questions (participants must answer ‘Yes’ to continue):**

*Are you a healthcare professional with experience delivering or organising palliative care interventions within the ICU?*

*Did the experience you are drawing from occur in the UK?*

*After reading the information sheet, please state whether you consent to completing the survey.*

**Survey introduction**

This survey asks questions about the implementation of providing palliative care within the intensive care unit. We understand that people involved with this have different roles, and that people may have more than one role.

From the statements below please choose an option that best describes your main role in relation to providing palliative care within the intensive care unit:

*I am involved in managing or organising palliative care within the intensive care unit*

*I am involved in providing palliative care within the intensive care unit*

For this survey, please answer all the statements from the perspective of this role. Depending on your role or responsibilities, some statements may be more relevant than others. For the purpose of this survey, the word “providing” will encompass managing, overseeing, or delivering palliative care withing the intensive care unit.

The survey is in 3 parts. Part A asks some brief questions about yourself and your role. Part B includes three general questions about providing palliative care within the intensive care unit. Part C contains a set of more detailed questions about providing palliative care within the intensive care unit. Please take the time to decide which answer best suits your experience for each statement.

This survey is adapted from: Finch et al., 2013. Improving the normalization of complex interventions: measure development based on normalization process theory (NoMAD): study protocol. Implementation Science 2013, 8:43.

**Adapted NoMAD Questions**

*When you are involved in providing palliative care in the ICU, how familiar does it feel (Likert scale 1-10)?*

*0 (still feels very new) to 10 (feels completely familiar)*

*Do you feel providing palliative care in the ICU is currently a normal part of your work (Likert scale 1-10)?*

*0 (not at all) to 10 (completely)*

*Do you feel providing palliative care in the ICU will become a normal part of your work in the future (Likert scale 1-10)?*

*0 (not at all) to 10 (completely)*

*For each statement, please select an answer that best suits your experience.*

*Strongly agree/Agree/Neither agree nor disagree/Disagree/Strongly disagree/Not relevant*

- I can see how providing palliative care in the ICU differs from usual ways of working
- Staff in this organisation have a shared understanding of the purpose of providing palliative care in the ICU
- I understand how providing palliative care in the ICU affects the nature of my own work
- I can see the potential value of providing palliative care in the ICU for my work
- There are key people who drive providing palliative care in the ICU forward and get others involved
- I believe that participating in providing palliative care in the ICU is a legitimate part of my role
- I’m open to working with colleagues in new ways to provide palliative care in the ICU
- I will continue to support providing palliative care in the ICU
- I can easily integrate providing palliative care in the ICU into my existing work
- Providing palliative care in the ICU disrupts working relationships
- I have confidence in other people’s ability to provide palliative care in the ICU
- Work is assigned to those with skills appropriate to provide palliative care in the ICU
- Sufficient training is provided to enable staff to implement providing palliative care in the ICU
- Sufficient resources are available to support providing palliative care in the ICU
- Management adequately supports providing palliative care in the ICU
- I am aware of reports about the effects of providing palliative care in the ICU
- The staff agree that providing palliative care in the ICU is worthwhile
- I value the effects that providing palliative care in the ICU has had on my work
- Feedback about providing palliative care in the ICU can be used to improve it in the future
- I can modify how I work to provide palliative care in the ICU

Single free-text question:

Would you like to tell us anything else about how you work to provide palliative care in your intensive care unit?
